# 

**Published:** 1960-10

**Authors:** 


MEDICAL JOURNAL OF THE SOUTH-WEST

*(Incorporating the Bristol Medico-Chirurgical Journal)*

Established 1883

VOL. 75 (iv) October 1960 NO. 278

### Contents

| | | |
|---|---|---|
| CLINICAL GENETICS AND THE PRACTITIONER . . . . . | *Thomas E. Oppe* | 77 |
| PAIN . . . . . . . . . | *Leslie Hill* | 89 |
| THE EVOLUTION OF PAEDIATRICS . . . | *John Apley* | 98 |
| A CASE OF MENINGEAL CARCINOMATIS . . . | *Clinical Pathological Conference* | 105 |
| NEWS FROM SOCIETIES . . . . . . . | | 108 |
| APPOINTMENTS . . . . . . . . | | 110 |

*PUBLISHED QUARTERLY*

1 *ANNUAL SUBSCRIPTION* 16/- *POST FREE*

PUBLISHED UNDER THE AUSPICES OF THE
BRISTOL MEDICO-CHIRURGICAL SOCIETY

### Editorial Committee

Dr. J. E. Cates, Department of Medicine, Bristol Royal Infirmary. *Editor.*
Dr. N. J. Brown, Southmead Hospital, Bristol. *Assistant Editor.*
Dr. A. B. Raper, Department of Pathology, Bristol Royal Infirmary. *Assistant Editor.*
Dr. J. Macrae, Ham Green Hospital.
Dr. R. C. Wofinden, Central Clinic, Bristol.
Mr. A. E. S. Roberts, Medical Library, University of Bristol. *Secretary.*

### *Local Correspondents*

*Bath* . . Mr. A. D. Bateman, 3 The Circus, Bath.
*Exeter* . . Dr. L. N. Jackson, Mount Jocelyn, Crediton, Devon.
*Gloucester* . Dr. R. F. Jarrett, 9 College Green, Gloucester.
*Plymouth* . Dr. C. R. Croft, Lexden, Hartley Av., Plymouth.
*Taunton* . Dr. M. McAlley, 1 The Crescent, Taunton.
*Torquay* . Dr. A. Robinson Thomas, 20 Devon Square, Newton Abbot, Devon.
*Truro* . . Dr. F. D. M. Hocking, St. Andrews, Perranporth, Cornwall.
*Yeovil* . . Mr. J. A. Vere Nicoll, Knapp House, Yeovil, Somerset.

### NOTICE TO CONTRIBUTORS

Original articles are invited, provided they have not been published elsewhere. Notes of forthcoming events, meetings held, degrees conferred, staff appointments, and other local news are welcomed. The editorial committee reserves the right to make literary corrections.

Illustrations should always be supplied ready for reproduction. All re-touchings or other operations required will be charged to the author. Line drawings should be drawn in Indian ink. We regret that we must ask authors to pay for making blocks, if this is necessary.

J. W. ARROWSMITH LTD., WINTERSTOKE ROAD,
BRISTOL.

### *Appointments*

### *SOUTH WESTERN REGIONAL HOSPITAL BOARD*

*March* 1960 *to June* 1960

### *BRISTOL*

| *Name* | *Appointment* | *Formerly* |
|---|---|---|
| PHILPOTT, B., M.B., B.S. (LOND.), D.A. | Registrar in Anaesthetics, Frenchay Hospital. | Anaesthetic Registrar-United Bristol Hospitals. |
| MCCLELLAND, H. A., M.B., B.S. (DUR'M). D.O.R.C.O.G., D.C.H. | Medical Registrar, Southmead Hospital. | Medical Registrar, Cheltenham General Hospital. |
| BROWSE, N. L. M.B., B.S. (LOND.), F.R.C.S. (Eng.). | Surgical Registrar, Southmead, Hospital. | Research Assistant to Professor R. Milnes Walker. |
| BURGESS, T. P., M.R.C.S., L.R.C.P. | Registrar in Psychiatry, Bristol Mental Group. | S.H.O. in Psychiatry, Barrow Hospital. |
| FLOOD, R. A., M.B., CH.B. (ST. ANDREWS) | Registrar in Psychiatry, Bristol Mental Group. | Registrar in Psychiatry, Bristol Mental Group. |
| GYAW, C. P., M.B., B.S. (RANGOON), D.A. (LOND.). | Anaesthetic Registrar, Southmead Hospital. | Locum Anaesthetic Registrar, Southmead Hospital. |
| MATHEWSON, HELEN S., M.B., CH.B. (ST. ANDREWS), D.P.M. | Registrar in Child Psychiatry & Mental Deficiency, Hortham-Brentry Group. | Registrar in Psychiatry, Prestwich Hosp., Nr. Manchester |

### *BATH*

| | | |
|---|---|---|
| ZAIDI, S. K. H., M.B., B.S. | Registrar in Anaesthetics to the Bath Group of Hospitals. | Resident Anaesthetist (S.H.O.) Epsom District Hospital. |
| CRAWFORD, J. W., M.B., CH.B. (GLAS.) D.O.R.C.O.G. | Registrar in Obst. & Gynae., Bath Group of Hospitals. | Registrar in Obstetrics, Forth Park Maternity Hospital. |
| DUCAT, E. F., M.B., CH.B. (ABERDEEN) | Clinical Assistant in Paediatrics, Royal United Hospital. | In general practice in Batheaston. Also present holder of Clinical Assistant post. |
| CHANDRASEKARAN, M. S. I. B.SC., M.B., B.S. (MADRAS), D.L.O. (LOND.) | Registrar in E.N.T. Surgery to the Bath Group of Hospitals. | E.N.T. Registrar, Bath Group of Hospitals. |
| HEAP, L. D., M.R.C.S., L.R.C.P. | Registrar in Pathology to Bath Group of Hospitals. | Resident Clinical Pathologist (S.H.O.), St. George's Hosp., London, S.W.1. |
| BURROWES, W. L., M.D., M.R.C.P | Clinical Assistant in General Medicine to the Bath Group of Hospitals, for one year. | Clinical Assistant in General Medicine to the Bath Group of Hospitals, and in general practice. |
| PINCHING, J., B.M., B.CH., M.R.C.P. | Clinical Assistant to Royal United Hospital, Bath. | Clinical Assistant to Royal United Hospital, Bath, and in general practice. |
| ELLISON, D. J., M.B., CH.B., M.R.C.P. | Clinical Assistant to Royal United Hospital, Bath, for one year. | Clinical Assistant to Royal United Hospital, Bath, and in general practice. |
| RONN, H. H., M.B., B.S., M.R.C.P. | Clinical Assistant to Royal United Hospital, Bath, for one year. | In general practice in Westbury, Wilts. |
| DUNBAR, P. G., M.B., B.S. (Lond.), D.A., D.OBST., R.C.O.G. | G.P. Anaesthetist, Devizes. | In general practice, Devizes. |

### NORTH GLOUCESTERSHIRE

| *Name* | *Appointment* | *Formerly* |
| --- | --- | --- |
| SOLTAU, D. H. K., M.A., M.B., B.CHIR.(CANTAB)., F.R.C.S.(ENG.), M.R.C.O.G. | Consultant Obstetrician and Gynaecologist to the North Gloucestershire Clinical Area. | Senior Registrar in Obstetrics and Gynaecology, St. George's Hospital and Southampton Group of Hospitals. |
| THOMSON D. S., M.B., B.S. (DUR'M.) D.O.M.S., F.R.C.S. (ED.). | Consultant Ophthalmic Surgeon to North Gloucestershire Clinical Area. | Senior Registrar, Oxford Eye Hospital. |
| DAVEY, R B., M.B., B.S. (SYDNEY) | Surgical Registrar, Gloucestershire Royal Hospital. | |
| MULLALLY, SHEILA, M.B., B.S., D.A. | Anaesthetic Sessions, Standish/Stroud. | In general practice combined with anaesthetic sessions at Cheltenham. |
| PARKER, SHEILA M., M.B., CH.B., (BIRMINGHAM), D.A., F.F.A., R.C.S. | Anaesthetic Sessions, Standish/Stroud. | In general practice in Stroud, Gloucestershire. |
| SHEPHARD, G. D. H., M.A., M.B., B.CHIR. (CANTAB.), D.OBST.R.C.O.G. | G.P. Anaesthetist, Tewkesbury Hospital. | In general practice in Tewkesbury. |
| GOMPERTZ, R. M. H. M.B., B.S. (Lond.), D.OBST.R.C.O.G., D.C.H. | Clinical Assistant in Paediatrics, Cheltenham Gen. and Maternity Hospitals. | In general practice in Cheltenham. |

### DEVON AND EXETER

| | | |
| --- | --- | --- |
| PRYER, D. L., M.B., B.S. (LOND.), F.F.A.R.C.S. | Consultant Anaesthetist to the Torbay Area. | Senior Registrar in Anaesthetics to the Oxford Regional Hospital Board. |
| GIBBS, D. F., CH.B. (ED.). M.R.C.P. (ED.) | Assistant Geriatrician (S.H.M.O.) to the Devon & Exeter Clinical Area. | Registrar, Lansdowne Hospital, Cardiff. |
| JEFFERIES, GILLIAN A., M.B., B.S. (LOND.), D.A. | Registrar in Anaesthetics, Royal Devon & Exeter Hospital. | S.H.O. in Anaesthetics, St. Mary's Hospital, Portsmouth. |
| SOUTHWOOD, W. F. W. M.B., B.CH. (CANTAB.), M.A.(CANTAB.), F.R.C.S. (ENG.), M.CHIR. (CANTAB.) | Senior Surgical Registrar (Joint appointment with United Bristol Hospitals). | Research Asst., St. Mark's Hospital, London. |
| COOPER, R. A., M.B., B.S. | Temporary G.P. Anaesthetist, Bideford Hospital. | General Practitioner, Bideford. |

### PLYMOUTH

| | | |
| --- | --- | --- |
| GORDON, D. T., M.B., CH.B., D.P.H. (ABERDEEN) | Senior Hospital Medical Officer in Infectious Diseases at the Scott Isolation Hospital, Plymouth. | Junior Hospital Medical Officer, Ham Green Hospital, Pill, Nr. Bristol. |
| CAM, J. F., M.B., B.S. (LOND.). | Registrar in Anaesthetics, South Devon & East Cornwall Hospital, Plymouth. | J.H.M.O., South Devon & East Cornwall Hospital. |
| COUTTS, J. P., B.SC. (N.Z.), M.B., CH.B.(N.Z.), F.R.C.S. (ENG.) | Surgical Registrar, South Devon & East Cornwall Hospital, Plymouth. | Locum Neurosurgical Registrar, Whittington Hospital, London. |
| MOSS, M. C., M.B., B.S., (ANDHRA) D.P.M. (CONJOINT) | Senior Registrar in Psychiatry, Moorhaven Hospital. | Psychiatric Registrar, Deva Hospital, Chester. |

### *CORNWALL*

| *Name* | *Appointment* | *Formerly* |
|---|---|---|
| ANDERSON, I. F., B.SC. (LOND.). M.R.C.S., L.R.C.P. | Registrar in Obstetrics and Gynaecology, Camborne-Redruth Hospital. | Locum Registrar in Obstetrics and Gynaecology, Camborne-Redruth Hospital. |
| BAIN, C. W. C., B.M., B.CH. (OXFORD), M.R.C.P., D.M., F.R.C.P. | Clinical Assistant in General Medicine, Royal Cornwall Infirmary. | Retired September, 1958, as Consultant Physician. |

### *1960 Index to Vol. 75*

Apply, J.—Evolution of Paediatrics, 98
Appointments—20, 41, 75, 110

Bourns, H. K.—Management of Sepsis, 50

Campbell, A. M. G.—Domestic Hazards, 23
Cardiac Ischaemia—G. Mather, 1.
Clinico-Pathological Conferences:
—Post partum necrosis of the Pituitary, 16
—Renal complications of Diabetes Mellitus, 37
—Nephrotic syndrome with Steatorrhoea, 64
—Meningeal Carcinomatosis, 105

Domestic Hazards of neurological significance—A. M. G. Campbell, 23

Examination Results, 41, 74

Genetics, clinical, for the Practitioner T. Oppé, 77

Hewer, T. F.—A Pathologist's survey of superficial tumours removed in a Casualty Department, 14

Hill, L.—Pain, 89
Hughes, F. W. T.—Mountains, 43

Kohn, F.—Semmelweiss and his times, 29

Mather, G.—Cardiac Ischaemia, 1
Middlemiss, J. H.—X-rays in everyday life, 55
Mountains—F. W. T. Hughes, 43

News from Societies, 20, 108
Notes and News, 20, 51, 74

Oppé, T.—Clinical Genetics and the Practitioner, 77

Paediatrics, the Evolution of—J. Apley, 98
Pain—L. Hill, 89

Semmelweiss and his times—F. Kohn, 29
Sepsis, the Management of—H. K. Bourns, 50

Tumours, superficial—T. F. Hewer, 14

X-rays in everyday life—J. H. Middlemiss, 55

